# Patterned photostimulation via visible-wavelength photonic probes for deep brain optogenetics

**DOI:** 10.1117/1.NPh.4.1.011002

**Published:** 2016-12-06

**Authors:** Eran Segev, Jacob Reimer, Laurent C. Moreaux, Trevor M. Fowler, Derrick Chi, Wesley D. Sacher, Maisie Lo, Karl Deisseroth, Andreas S. Tolias, Andrei Faraon, Michael L. Roukes

**Affiliations:** a Kavli Nanoscience Institute, California Institute of Technology, Pasadena, California 91125, United States; b California Institute of Technology, Departments of Physics, Applied Physics, and Bioengineering, 1200 East California Boulevard, MC149-33, Pasadena, California 91125, United States; c Baylor College of Medicine, Department of Neuroscience, One Baylor Plaza, Suite S553, Houston, Texas 77030, United States; d Stanford University, Department of Bioengineering, Stanford, West 250, Clark Center, 318 Campus Drive West, California 94305, United States; e Stanford University, Howard Hughes Medical Institute, Department of Psychiatry and Behavioral Sciences, West 083, Clark Center, 318 Campus Drive West, Stanford, California 94305, United States; f California Institute of Technology, Departments of Applied Physics and Medical Engineering, 1200 East California Boulevard, MC107-81, Pasadena, California 91125, United States

**Keywords:** optogenetics, photonic probes, visible photonics

## Abstract

Optogenetic methods developed over the past decade enable unprecedented optical activation and silencing of specific neuronal cell types. However, light scattering in neural tissue precludes illuminating areas deep within the brain via free-space optics; this has impeded employing optogenetics universally. Here, we report an approach surmounting this significant limitation. We realize implantable, ultranarrow, silicon-based photonic probes enabling the delivery of complex illumination patterns deep within brain tissue. Our approach combines methods from integrated nanophotonics and microelectromechanical systems, to yield photonic probes that are robust, scalable, and readily producible *en masse*. Their minute cross sections minimize tissue displacement upon probe implantation. We functionally validate one probe design *in vivo* with mice expressing channelrhodopsin-2. Highly local optogenetic neural activation is demonstrated by recording the induced response—both by extracellular electrical recordings in the hippocampus and by two-photon functional imaging in the cortex of mice coexpressing GCaMP6.

## 1 Introduction

An overarching technological goal in the field of optogenetics is the development of new methods for stimulating neural circuits with very high spatiotemporal precision. Ongoing efforts seek to address large functional ensembles of neurons, i.e., “brain circuits,” through realization of tools providing fine enough resolution to interrogate and control each constituent neuron individually and independently. Significant advances in the development of excitatory and inhibitory opsins have been made over the past decade that now permit direct optical control of cellular processes.^1^ To realize the full potential of these technologies, complementary methods for delivering light with cellular precision *in vivo* are now essential.^2^^,^^3^ Existing, state-of-the-art approaches involve the use of spatially patterned light, projected via free-space optics, to stimulate small and transparent organisms^4^^–^^6^ or excite neurons within superficial layers of the cortex.^7^^,^^8^ However, light scattering and absorption in neural tissue, characterized by the optical attenuation length, cause ballistic light penetration to be extremely short.^9^ This makes it impossible to employ free-space optical methods to probe brain regions deeper than about ∼2  mm. This statement holds true even if we take into account methods for two-photon and three-photon excitation, and recent efforts made to develop opsins that operate in the red or near infrared. With these limitations in mind, we advance here an alternative approach, involving implantable photonic devices, as the most promising paradigm for delivering and projecting high-resolution patterned light at “arbitrary depths” and with minimal perturbation in the brain.

We identify five critical requirements for realizing widely useful, implantable photonic devices, which we term “visible-wavelength photonic probes”: (i) the probes should provide a multiplicity of microscale illumination sources (hereafter “emitter pixels,” or “E-pixels”), each individually controllable and capable of delivering fine illumination, with cellular-scale cross-section dimensions. Ideally, emission from these microscale E-pixels should have minimal spatial overlap, while collectively covering the entire brain volume of interest. (ii) This patterned illumination must be delivered with sufficient intensity to activate optogenetic effectors (actuators/silencers) within the interrogated region. (iii) Associated thermal perturbations of neural tissue at, or adjacent to, the implanted devices must minimally affect neural circuits. Recent studies show that temperature elevation of as small as 1°C can change the neural firing rate and behavior of mice.^10^ (iv) The cross-sectional dimensions of the probes must be made as small as possible—to reduce displacement of brain tissue upon implantation, to minimize tissue damage, and to suppress potential immunological response.^11^ (v) Finally, photonic nanoprobe fabrication should be compatible with, and ultimately transferrable to, foundry (factory)-based methods for mass production. This will permit wide deployment of this new technology in the near-term to the neuroscience community. Here, we present a class of photonic probes satisfying these requirements; they are based on integrated, silicon-based nanophotonic components adapted to operate at visible wavelengths and embedded onto implantable silicon probes patterned by microelectromechanical systems (MEMS) processes.

## 2 Implantable Photonic Neural Probes

Various architectures for implantable optical probes have recently been proposed.^12^ For example, one approach relies upon multiple optical fibers to excite individually addressed illumination points, each driven by a dedicated laser source.^13^^,^^14^ Given the complexity of coupling many fibers to a probe, this approach is capable of providing only a few illumination points. To surmount this issue, coupling a fiber-bundle to on-chip photonic waveguides has been proposed,^15^ but neither *in vitro* nor *in vivo* validation of this particular approach has been reported. Another approach implements modal multiplexing to address several illumination points along an implantable multimode optical fiber.^16^ However, this approach necessitates a rather large distance between illumination points (>200  μm) to avoid overlap between adjacent illumination beams. Neither approach is readily upscalable to many emission points, nor easily produced *en masse*.

An alternative approach involves the integration of microscale light emitting diodes (μLEDs) directly onto the probe shanks.^17^^–^^19^ A variation on this theme integrates laser diodes upon the probe head,^20^^–^^22^ with their light output routed by on-chip integrated photonic waveguides to emission points located along the shanks. In both cases, however, the power dissipated by these active μLED devices must be strictly limited, given that neuronal activity thresholds are highly sensitive to minute temperature variations.^10^^,^^23^ Minimizing the total heat delivered to brain tissue by the probe, which is dominated by the heat generated by the μLEDs in these architectures, can significantly restrict the number of active illumination sources that can be integrated. Unless the efficiency of μLED or laser diode sources is dramatically increased, it will not be feasible to include more than a limited number of active light emitters on implantable photonic probes.

Here, we present a new paradigm for photonic probes that employs wavelength division multiplexing (WDM).^24^ It provides the potential for massive upscaling of the number of E-pixels that can be incorporated and individually addressed within implantable, ultra-compact neural probes. The technique of WDM employs a multiplicity of independent data streams, each imprinted on individual carrier wavelengths (spectral channels), that are combined (i.e., multiplexed) and transmitted via a single optical fiber. At the receiving end, these multispectral signals are subsequently demultiplexed and delivered to their intended destinations. In our application of WDM, each temporally modulated carrier wavelength is delivered to an independent E-pixel at a specific, spatial location located along an implantable photonic probe shank. Spectral separation is achieved by photonic circuitry for WDM integrated within the probe head. Our technique is exceptionally well suited for optogenetic effectors, because currently employed opsins respond to a relatively broad spectrum of light, typically spanning ∼50  nm.^25^^,^^26^ This permits accommodating many spectral channels within the opsin absorption band. Additionally, this unique assignment of different wavelengths to specifically located E-pixels can be accomplished solely using “passive” components, which neither requires power to operate, nor generate additional heat.

The photonic neural probes described herein provide a first proof-of-concept of our paradigm. The prototype devices we report here comprise implantable shanks, initially with nine E-pixels, which are spectrally addressed through “one” single-mode optical fiber. We implement the E-pixels themselves using large diffractive grating couplers that produce beams with low divergence angles, as small as 1.7 deg. This low-light divergence offers beam cross-section dimensions that are comparable to the size of neural cell bodies—even after traversing several hundreds of micrometers. Other recent implementations of implantable probes based on photonic technology^27^ do not provide a route toward the goal of generating complex illumination patterns with narrow illumination beams at arbitrary locations within the brain.

## 3 Probe Architecture and Fabrication

**Fig. 1:**
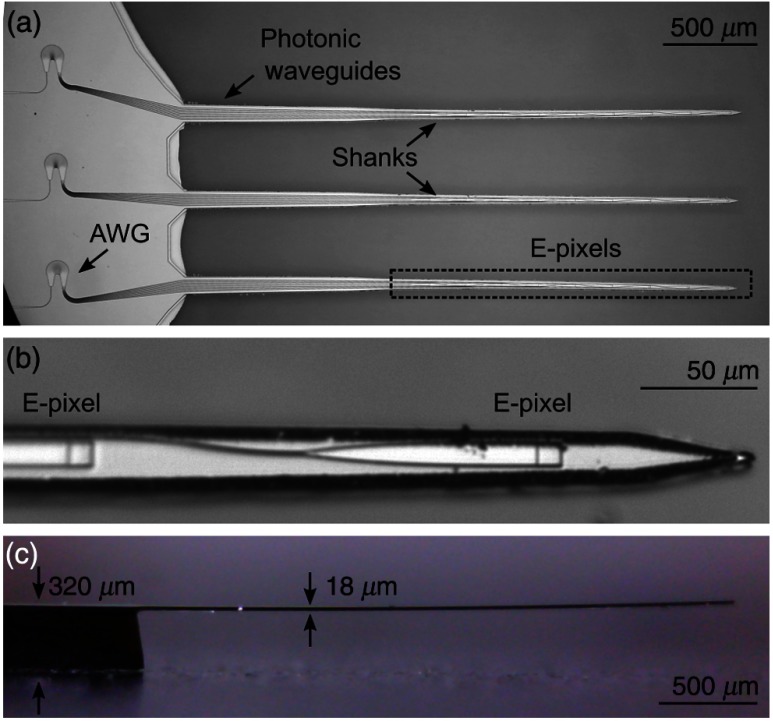
Prototype photonic probe architecture. (a) Optical micrograph showing photonic probes operating at visible wavelengths. This specific design contains three 3-mm-long, 18-μm-thick shanks that taper in width from 90  μm, at the head, down to 20  μm, near the tip. The photonic elements comprise three AWG demultiplexers; one per shank. Each is driven by a single-input waveguide (left) and subsequently drives a multiplicity of output waveguides (right) that traverse the shanks, carrying light to their ultimate destinations on the shank tips. At their termini, the photonic waveguides drive grating couplers (termed E-pixels), which couple light off-shank into brain tissue. All the on-chip photonic elements are patterned from a 200-nm thick silicon nitride layer, which is deposited on top of the oxidized silicon structural layer used to form the probe body. (b) Photograph showing the top view of two 10  μm×10  μm grating couplers that constitute the E-pixels near the tip of a shank. The tapered waveguides transform the small optical cross section of the submicron waveguides to the larger neuronal-scale spot size delivered by the E-pixels. (c) Side view of a photonic probe. Although the shank thickness is 18  μm, the thicker ∼320  μm probe head (on left) facilitates handling and mounting of the device.

The overall structure of our prototype photonic probes is patterned using standard nanophotonic and MEMS fabrication processes (Appendix A, Fig. 6). The implantable, needle-like probe shanks have widths of ∼90  μm near the probe head decreasing to only ∼20  μm near the tip, with a uniform thickness of 18  μm throughout. The shank tips are wedge-shaped [Fig. 1(b)], with tips having a ∼1  μm radius of curvature; this ensures smooth penetration of brain tissue with minimal dimpling.^28^ Our approach yields implantable probes with overall cross sections representing the state-of-the-art for optical probes. They are far smaller than the optical fibers or endoscopes currently implanted for optogenetic experiments (Appendix A, Fig. 9). The shanks of the prototype probes reported here have a pitch of 200  μm and lengths of either 3 or 5 mm, yet they remain straight after fabrication through our careful engineering of the ubiquitous internal stresses present within thin-film multilayers (Appendix A).

The probe head [Fig. 1(a)] contains integrated nanophotonic circuitry required to couple multispectral light delivered from a single external optical fiber onto the probe chip and, subsequently, to route the individual spectral components (channels) to specific emitters on the shank(s). E-pixels arrays [Fig. 1(b)] can be placed at any location along the implantable shanks; in the first prototypes reported here, we include nine E-pixels, spaced on a 200-μm pitch. It is straightforward to achieve ≤50  μm spacing between adjacent E-pixels without changing our fabrication protocols^29^ (Appendix A).

## 4 Nanophotonic Circuitry

**Fig. 2:**
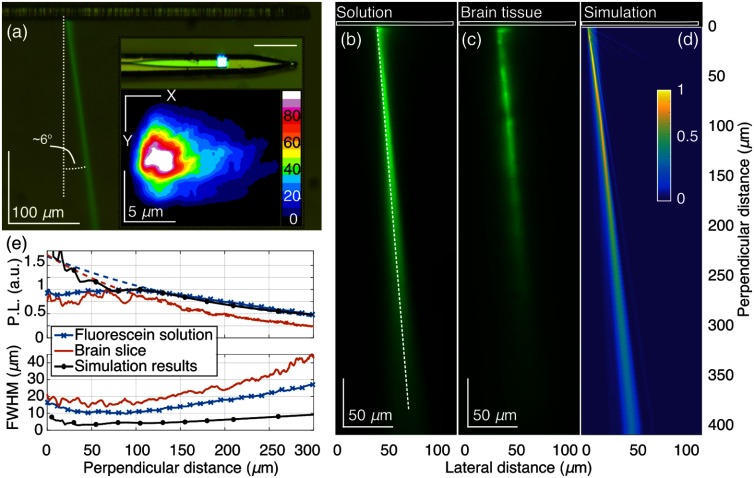
Characterization of E-pixel illumination. (a) Optical micrograph showing the side view of a shank immersed in a fluorescein–water solution to visualize the E-pixel illumination profiles. In this image, blue light (473 nm) is emitted from an E-pixel located ∼250  μm away from the tip of the shank. This light stimulates the green photoluminescence (PL) visible in the image. (Inset, top) Optical micrograph showing another E-pixel (here, ∼100  μm from the shank tip) emitting blue light. Scale-bar corresponds to 50  μm. (Inset, bottom) Normalized iso-intensity contours for light measured at the surface of an E-pixel. (b) and (c) Measured green PL intensity pattern, covering a distance of 410  μm, generated by the blue (473 nm) illumination beam emitted by the photonic E-pixel. Image (b) was obtained in a 10-μM fluorescein–water solution at pH>9.5; image (c) was obtained from a fluorescein-stained mouse brain slice. The dashed line delineates the beam trajectory. (d) Simulated E-pixel illumination intensity profile in water. Results were obtained using the nominal probe design parameters. (e) PL beam profile analysis. (Top) Continuous lines correspond to measured and simulated normalized PL intensity, calculated along the beam trajectory as a function of distance from the E-pixel. Normalization of the experimental and simulation result is done relative to the maximum beam intensity (measured at a distance of 70 to 100  μm) and the simulated intensity at a distance of 100  μm, respectively. Dashed lines show fit results of the far-field intensity to an exponential decaying function. (Bottom) Analysis of the beam width (FWHM, i.e., full width at half maximum) as a function of the distance from the E-pixel.

The visible-wavelength photonic circuitry on the probe is fabricated from an optical multilayer comprising a 200-nm thick silicon nitride (${\mathrm{Si}}_{3}N_{4}$) layer encapsulated between two layers of silicon dioxide (${\mathrm{SiO}}_{2}$), to yield a total thickness of 2.8  μm. This multilayer is grown upon a commercially available silicon-on-insulator (SOI) substrate, itself comprising a 15-μm-thick Si (structural) layer atop, a 2-μm buried oxide (BOX) layer, above a 300-μm-thick Si wafer. The photonic circuit is comprised of grating couplers, photonic waveguides, and arrayed waveguide gratings (AWGs). In Appendix A, we describe a microprism coupling method (hereafter, *μ*-prism) bridging the external input fiber’s terminus to the on-chip grating coupler. This efficiently couples the light to the photonic waveguides on-chip. Once on-chip, this multispectral light is routed by a single waveguide to an AWG located on the probe head [Fig. 1(a)]. The AWGs function as passive optical demultiplexers that spectrally separate the incoming wavelength-multiplexed signal to the nine output waveguides. These separated post-AWG signals are subsequently routed by separate photonic waveguides onto the shank, and then to their termini at individual E-pixels. These E-pixels (described in Appendix B) comprise small-footprint diffractive grating couplers patterned on the surface of the shanks [Fig. 2(a) inset]; light routed to each is emitted off-shank, almost perpendicularly, into adjacent neural tissue. Our prototype devices require a single optical fiber per AWG or shank. Future designs will incorporate a hierarchical on-probe photonic circuit, in which a master AWG drives subsequent AWGs, to reduce the total number of required optical fibers to one.

The critical integrated photonic elements on the probes—the AWGs and the grating couplers—require spatiotemporally coherent light for their operation. We drive them with multispectral light generated and modulated off-probe, and then delivered to the probe head by a single external optical fiber. The ratio of the incident power delivered by the fiber, to the total power emitted by the E-pixels, defines the probe insertion loss (IL). The total IL of these first unoptimized prototypes is about ∼20  dB. Roughly, ∼16  dB of this arises from coupling loss into and out of the probe, dominated by nonideal coupling between the fiber and the on-chip photonic circuitry. The various losses present are fully delineated in Appendix B, Fig. 11. In future device generations, these ILs can be reduced significantly through advanced engineering design and, especially, by use of the highly optimized fabrication processes available at commercial photonic foundries.^30^ We emphasize that the majority of these losses, 18 dB in our current prototypes occurs within the probe base, rather than at the point of emission, as is the case for μLEDs.

Validation of the capability of our photonic probes to stimulate neural activity, as described in the next section, has been achieved with E-pixel emission power that ranged between 5 to 10  μW. With IL of 20 dB, incident laser power of 1 mW per E-pixel is required. Such power is readily available over the relevant wavelength range with supercontinuum lasers.

## 5 Characterization of Single E-Pixel Illumination

Our measurement and simulations results demonstrate that E-pixels emit beams with a propagation direction angle of 2 to 30 deg from the normal to the probe surface [Fig. 2(a)]. The exact angle of each individual E-pixel can be engineered during the probe design phase by setting the period of the grating couplers. Once probes are fabricated, this angle is fixed. The low divergence of the beams minimizes overlap between adjacent beams, while preserving light intensity over significant propagation distances from the E-pixel. We have capitalized on the highly collimated photonic probe beamshape to enable local optogenetic activation of neurons.

The beam profile at the surface of the E-pixel is <6  μm (FWHM) along both transverse axes of the beam [Fig. 2(a) inset]. We characterize the beam profile versus distance from probe shank by: (i) imaging in a fluorescein solution [Figs. 2(b) and 13], (ii) imaging in, ∼300-μm thick, adult mouse brain slices soaked overnight in a fluorescein solution [Fig. 2(c)], and (iii) comparison with numerical simulations [Figs. 2(d), 14, and 15]. We find Fresnel diffraction determines the beam intensity profile up to a distance of ∼70  μm from the probe; beyond that, it is characterized by far-field Fraunhofer diffraction. (Appendix B; Fig. 15). The minimal beam width, observed at the transition between the Fresnel and far-field regions at a distance of ∼90  μm [Fig. 2(d)], is ∼10  μm in the fluorescein solution, ∼17  μm at a distance of ∼70  μm in the brain slice, and <5  μm at distances smaller than 100  μm in our simulations. Light scattering results in a slightly larger beam divergence in tissue [Fig. 2(c)] than in the fluorescein solution [Fig. 2(b)]. However, all beam widths measured up to a distance of 200  μm are less than, or of order, the size of an individual neuronal cell body. This property of E-pixel illumination permits reducing the E-pixel pitch to ≤50  μm, while still maintaining negligible overlap between adjacent beams.

## 6 Multibeam Illumination

**Fig. 3:**
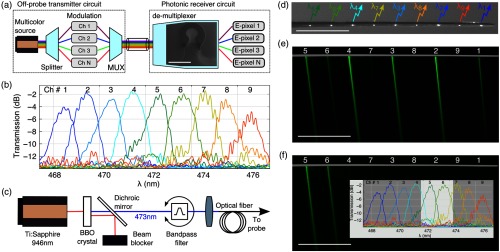
Multibeam illumination. (a) Schematic elucidating the concept of WDM applied to photonic neural probes. The inset in the demultiplexer block shows an electron micrograph of one of our blue-wavelength AWGs (the scale bar represents 100  μm; Fig. 12). (b) Transmission measurements of the various output channels of the AWG versus input light wavelength. Measurements are obtained by delivering a broadband light input to the AWG and measuring the output spectrum emitted from each output channel with a spectrometer. (c) Schematic showing the optical setup used to address individual channels of the AWG. We employ a Ti:Sapphire pulsed laser (946 nm) to pump a BBO crystal, which generates blue (473 nm) light by exploiting a second harmonic generation process. Unconverted infrared light is redirected into a beam blocker by a dichroic mirror; the converted blue light is coupled to the optical fiber. The 13-nm bandwidth of the infrared pump signal reduces, after conversion, to blue light with a bandwidth of ∼5.5  nm. This is subsequently narrowed spectrally with a manually tuned bandpass filter (Alluxa, FWHM 1 nm). Rotation of the filter tunes the central wavelength of this filter, thereby enabling dynamic tuning of the passband. A shutter or an acousto-optic modulator can be added at any point along the beam to temporally modulate the light. (d) Optical micrograph showing a shank with nine simultaneously driven E-pixels. The center-to-center pitch between E-pixels is 200  μm in these prototypes. The annotations denote the mapping between the spectral channels plotted in panel (b) and the spatial location of the corresponding E-pixels to which they are coupled. The scale bar here represents 500  μm. (e) and (f) Measured fluorescein PL patterns generated by simultaneous illumination from several E-pixels. The spectrum (bandwidth and central wavelength) was set to 5.5 nm at 470 nm, and 1.8 nm at 473 nm, in panels (e) and (f), respectively. The inset in panel (f) superimposes this spectrum with the spectral response of the AWG. Here, scale bars represent 500  μm.

Our use of WDM makes it possible to independently address on-shank E-pixels by separate temporally modulated multispectral components of the light delivered to the probe. Figure 3(a) shows an illustration of our WDM approach. Coherent light from a broadband (multispectral) source is split into N discrete spectral bins; each is employed as an independently controllable transmission channel. Temporal modulation providing the unique, arbitrarily complex illumination pattern required for simultaneous excitation of specific locations within the brain is imposed on each of these spectral channels. Figure 16 depicts a possible approach for temporal modulation, using of the shelf components.

Our embodiment of the on-chip optical demultiplexer is realized with a visible wavelength AWG [Fig. 3(a), inset; Fig. 12]. AWGs are now perfected and commercially available for use with infrared light in telecommunications technology,^31^ however, given their need for much tighter dimensional tolerances and smoother structures (to suppress diffuse sidewall scattering),^32^^–^^34^ there are only a couple of reports of AWGs configured for the visible spectrum to date. The blue-wavelength AWGs we have developed for this work have a compact footprint of <150  μm×150  μm; accordingly, they are ideally suited for integration within the heads of our miniature photonic probes [Fig. 1(b)]. By appropriately synthesizing the multispectral light input [Figs. 3(b) and 3(c)], either individual E-pixels or a multiplicity of them can be independently and simultaneously addressed [Figs. 3(e) and 3(f)]. Figure 16 presents a schematic of one possible setup for addressing E-pixels, which provides high temporal bandwidth using a tunable acousto-optic filter.

The maximum number of E-pixels addressable by a single AWG is determined by the ratio of the absorption bandwidth of the optogenetic effector to the bandwidth of the individual spectral channels. For example, a typical optogenetic effector such as ChR2^25^ has an absorption bandwidth of ∼50  nm centered near a wavelength of 460 nm, whereas the spectral channel width, an engineerable parameter, can be much narrower (Appendix C). In the designs here, we set the latter to ∼1  nm [Fig. 3(b)], thus permitting each AWGs to address up to ∼50 E-pixels^35^ with only a single external fiber input to the chip. Upscaling this number to even more E-pixels outputs per fiber input is readily achievable with precise foundry-based fabrication methods, which can permit definition of spectral channels with a roughly 10× narrower bandwidth. However, one must keep in mind that upscaling spectral channel density must be accompanied by a proportional increase in applied laser power per unit bandwidth, as the light will be distributed over a larger number of E-pixels within the effector’s absorption band.

## 7 Functional Validation

**Fig. 4:**
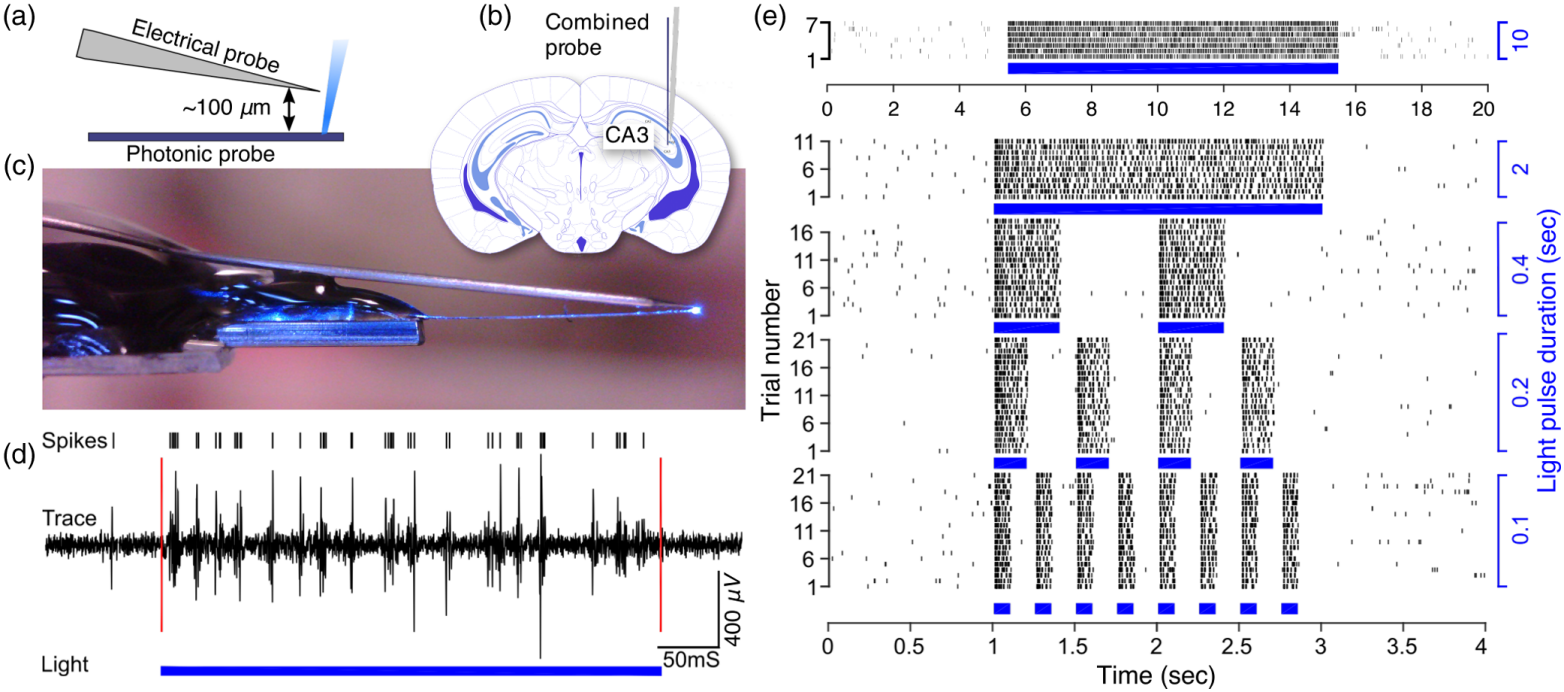
*In vivo* photoactivation of hippocampal CA3 pyramidal neurons in a mouse with concomitant electrophysiological recording. (a) Schematic depicting the relative configuration of a recording electrode tip, the photonic probe, and light that is emitted from one E-pixel. (b) The combined probe setup that is carefully implanted into the mouse brain within the CA3 region of the hippocampus. Optical emission from the E-pixel is directed toward the anterior region of the brain. (c) Photograph of the electrical probe (A-M Systems, tungsten electrode, d=127  μm, R=1  MΩ), which is affixed immediately above the photonic probe. The probe shank length is 3 mm. (d) Recordings obtained with the electrical probe, showing the response evoked from a 400-ms long optical excitation pulse (blue bar). Black lines above the recording denote spikes. Photoelectric transients, which occur at the location of the red lines, are generated when the optical excitation is switched on and off; these artifacts have been removed from the data for clarity. (e) Raster plot showing the spiking response evoked during repeated illumination trials, demonstrating the activation of ChR2 by blue light from the E-pixel. Several illumination patterns were tested, including 100-, 200-, and 400-ms long pulses (blue bars) with repetition rates of 4, 2, 1 Hz, respectively, as well as 2- and 10-s long pulses.

We validate the functional capabilities of our prototype photonic probes *in vivo*, in two separate experimental implementations. In the first, we optogenetically activate neurons in the hippocampus of Thy1:18-ChR2-EYFP transgenic mice,^36^ while simultaneously recording induced extracellular electrical activity close to the point of light stimulation. To achieve this, an electrically insulated tungsten wire was glued directly atop the photonic probe [Figs. 4(a) and 4(c)]. The uninsulated distal electrode tip is positioned 100  μm above the E-pixel. This composite probe was then advanced into the CA3 region of the hippocampus of an anesthetized, head-fixed mouse [Fig. 4(c)]. In this brain region, pyramidal neurons express high levels of ChR2-EYFP.^36^ Shortly after implantation, the illumination beam was directed rostrally and electrical measurements were recorded in response to optical stimulation pulses. Several temporal patterns of illumination were tested. The corresponding extracellular electrical recording [Figs. 4(d) and 4(e)] reveals repeated and intense multiunit spiking activity in direct response to light pulses delivered by the photonic probe. No significant degradation in the rate or amplitude is observed for these multiunit bursts, or even for pulses as long as 10 s. In these experiments, we estimate the optical power emitted by the E-pixel to be <5  μW.

**Fig. 5:**
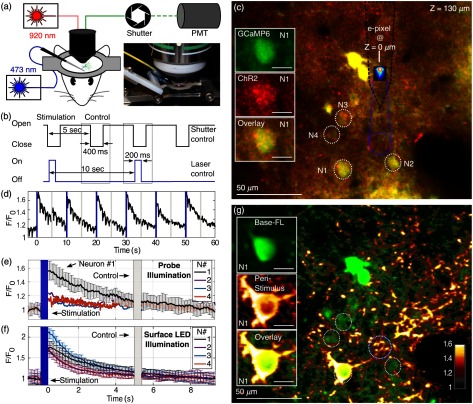
Cortical neural stimulation with concomitant two-photon optical functional imaging. (a) Schematic of the experimental setup. Measurements are carried out in an anesthetized, head-fixed mouse, placed under a custom two-photon microscope. The photonic probe is implanted at an angle of 35 deg relative to the surface of the optical dissection, providing access to the brain through the narrow gap between the microscope objective and the surgical opening. A fiber-coupled 473-nm diode laser drives the E-pixel at the tip of the photonic probe. To prevent saturation of the PMTs during application of optical stimuli, mechanical shutters are used to block light emitted by the probe. The inset shows a photograph of the experimental configuration. The narrow profile of the probe enables it to fit under the microscope objective. (b) Illustration of the light excitation sequence. Each sequence includes a single-stimulation event and a subsequent control event, during which only the mechanical shutter is activated while no light is emitted by the probe. This precaution permits identifying the level of response evoked by auditory stimuli; no response is observed. (c) Visualization of the expression levels of optogenetic actuators and reporters of the imaging site in mouse cortical layer 2/3 (920 nm excitation, Nikon 16×/0.8-NA objective; scale bar represents 50  μm). By overlaying two-photon photometry images, it is possible to identify neurons coexpressing both GCaMP6s and ChR2-mCherry (insets; scale bars represent 10  μm). This imaging site is located ∼130  μm above the tip of the probe, whose lateral position is marked by the dashed black line. The dashed blue circle marks the approximate probe beam position and width at the imaging plane of the microscope. The four dashed white circles, labeled N1 to N4, delineate four coexpressing neurons located in close proximity to the illumination site. (d)–(f) Results from neural excitation. (d) ${\mathrm{Ca}}^{2+}$ transients, measured for neuron #1, showing evoked neural response during sequential excitation and control events, as marked by blue and gray lines, respectively. Traces are normalized by the base fluorescence level, integrated over the first 30 ms prior to the stimuli event. (e) Peristimulus time histogram of neurons 1 to 4 calculated over 19 stimulation cycles. (f) Peristimulus time histogram of neurons 1 to 4 calculated over 15 pulses of wide-field blue (473 nm) illumination delivered through the microscope objective. (g) An overlaid image showing the peristimulus activation calculated at single pixel resolution, across the entire imaging site. Baseline fluorescence in green is calculated over a period of 40 ms prior to the stimuli. The black–red–yellow layer shows the peak peristimulus fluorescence, calculated as an average over a 30 ms interval following the stimulus event. The dashed circle marks are duplicated from panel b.

**Fig. 6:**
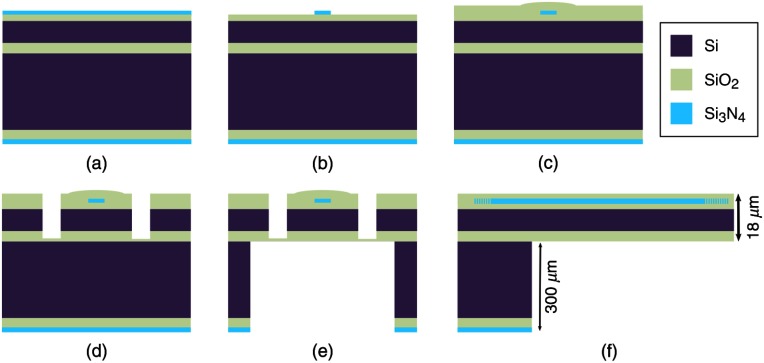
Schematic of the fabrication process flow. (a) Schematic of the SOI wafer after deposition of the ${\mathrm{Si}}_{3}N_{4}$ photonic layer. (b) Photonic circuitry patterning using electron-beam lithography and pseudo-Bosch etch processes. (c) Encapsulation of the photonic layer by deposition of PECVD ${\mathrm{SiO}}_{2}$. (d) Photonic probe front side patterning using photolithography and ICP etch processes. Front (e) and side (f) views of the photonic probe after the backside etch but before the HF etch step that removes the thin BOX remaining between the shanks.

**Fig. 9:**
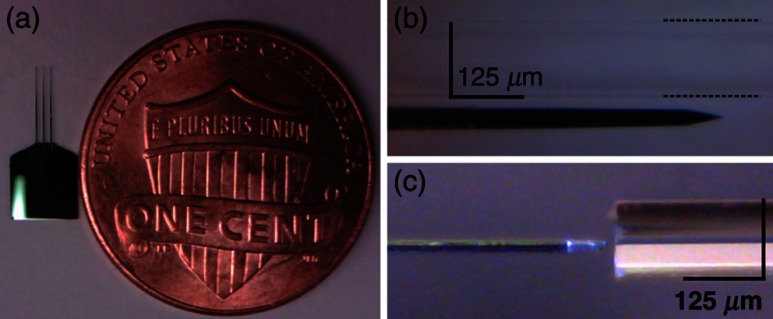
Probe dimensions. (a) Photograph showing the relative size of a probe having 5-mm long shanks compared to a U.S. penny. Optical micrographs showing (b) top and (c) side views of the tip of the shank compared to a 125-μm diameter optical fiber.

**Fig. 11:**
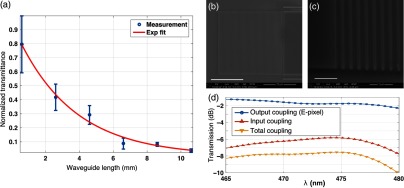
(a) Normalized waveguide transmittance as a function of the waveguide length. Error bars represent the standard deviation of the measured transmittance within each group of six waveguides. (b) and (d) Grating coupler images and simulated spectra. (b) and (c) Scanning electron microscope (SEM) images of an input grating coupler. The grating couplers have dimensions of 25  μm×25  μm. Image (c) was obtained at an angle of 52 deg to better visualize the partial etch of the ${\mathrm{Si}}_{3}N_{4}$ photonic layer between the grating teeth. (d) Finite-difference time-domain (FDTD) simulation results showing the transmission spectra of an improved input grating coupler using an in-line angle-polished fiber for coupling^40^^,^^41^ and the designed E-pixel (output grating coupler) used in the probes demonstrated in this work. The input grating coupler spectrum is the transmission between the fiber mode and the fundamental transverse-electric (TE) mode of the waveguide with a coupling angle of 5 deg; the E-pixel spectrum is the percentage of optical power radiated toward brain tissue with a fundamental TE mode input to the waveguide.

**Fig. 12:**
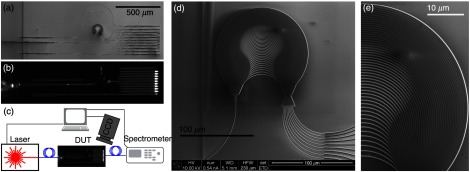
AWG measurement setup. (a) SEM image of an AWG test chip. The image shows an AWG having one input channel on the left and nine output channels on the right. Above and below the AWG are two waveguides used to de-embed the fiber-to-chip coupling losses from the AWG measurement. (b) Optical microscope image of the AWG, obtained while the AWG is excited with broadband multicolor light, thus all its output channels emit simultaneously. (c) Schematic of the optical setup used to measure the AWG. (d) and (e) SEM images of one of the AWGs designed to route blue light. This array is composed of ∼50 waveguides, each having a width of 240 nm.

**Fig. 13:**
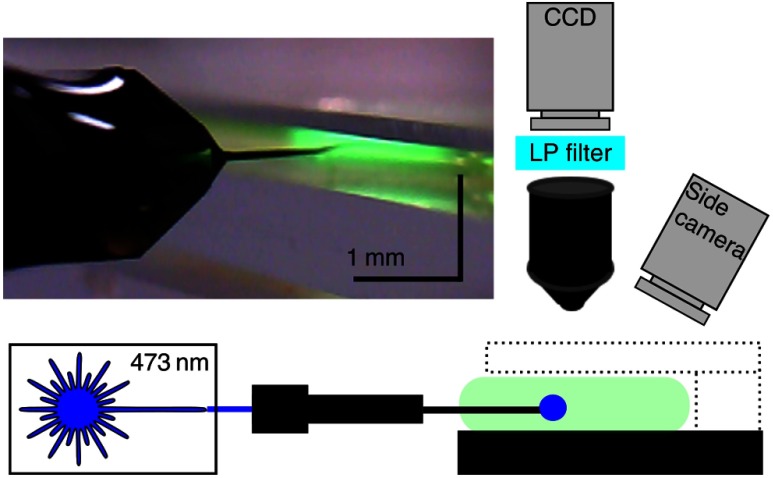
Fluorescein imaging setup. The photonic nanoprobe is inserted sideways into a small reservoir containing a fluorescein–water droplet. The probe is positioned vertically to be as close as possible to the top coverslip; this minimizes scattering along the imaging axis. (Inset) Photograph showing insertion of the probe into the fluorescein–water solution.

**Fig. 14:**
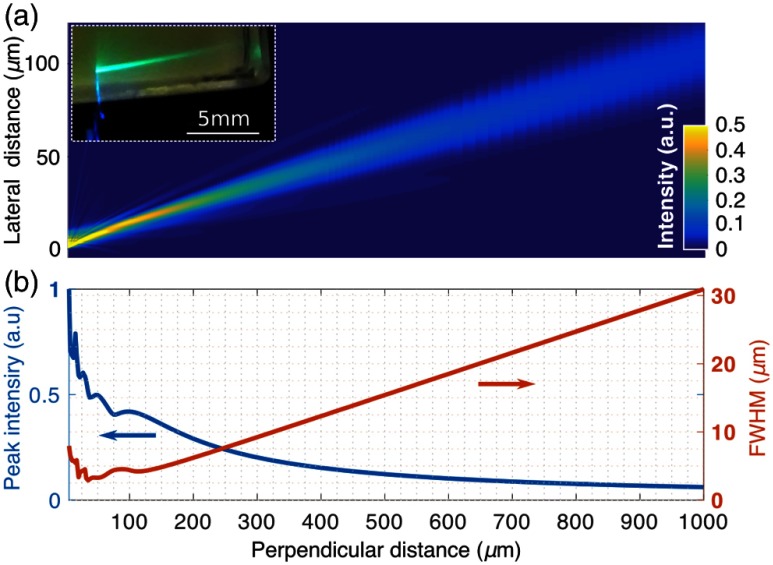
Far-field beam illumination pattern. (a) Simulation showing the emission beam intensity pattern spanning a distance of 1 mm from an E-pixel, which is implemented as an output grating coupler. The intensity was truncated at a value of 0.5 to improve image contrast. The inset shows a photograph of the beam shape in fluorescein. Image was taken using the setup depicted in Fig. 13, inset. Beam extends over a distance of almost 10 mm. (b) Analysis of the simulated beam in panel (a). Blue and red curves show the peak intensity and the FWHM transverse beam width, respectively, as a function of the distance away from the grating coupler.

**Fig. 15:**
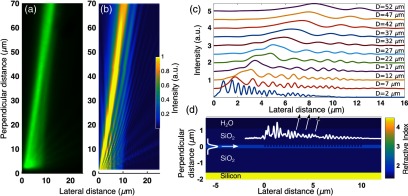
Fresnel diffraction pattern. (a) Optical image showing the green PL intensity pattern of the E-pixel emission. This image magnifies an area of 25  μm×70  μm close to the E-pixel, where the pattern is dominated by Fresnel diffraction. The weak illumination beams emitted at large angles are generated by the diffraction of light that is reflected back from the bottom silicon substrate (panel d). (b) Color map and (c) line-plot showing FDTD simulation results of the corresponding E-pixel normalized intensity emission pattern as a function of the lateral and perpendicular distances from the origin of the grating couplers. Lines in panel (c) are spaced by half a unit for clarity. (d) Color map showing the refractive indexes of the structure, calculated numerically by FTDT. The plotted white line shows the near-field emission intensity pattern calculated 0.5  μm above the grating structure.

**Fig. 16:**
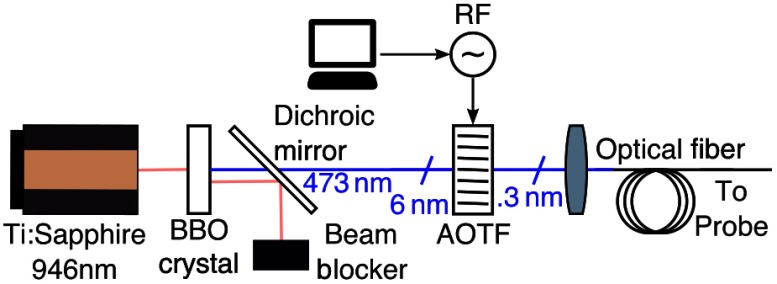
WDM setup. Schematic of a WDM setup based on an AOTF.

In a second experimental implementation, the functionality of the photonic probes was assessed via simultaneous free-space, two-photon functional imaging of cortical neurons in a mouse coexpressing both ChR2 and GCaMP6s (Fig. 5). The photonic probe was inserted at an angle of ∼35  deg into cortical layer 2/3. Although minor dimpling was observed during photonic probe insertion at this angle, the probe was sufficiently sharp to penetrate the dura with only moderate pressure. Probe illumination was directed upward from the surface of the probe into the brain tissue and a local population of neurons was imaged ∼130  μm above the probe tip [dashed white circles in Figs. 5(c) and 5(g)]. The approximate FWHM beam width at the imaging plane was ∼20  μm.

For this second set of experiments, 200-ms light pulses with an estimated optical output power of ∼10  μW were delivered by the photonic probe with a repetition rate of 0.2 Hz. The fluorescence response of the ChR2/GCaMP6s-expressing cells was simultaneously recorded by two-photon imaging. To prevent saturation of the photomultiplier tubes (PMTs) during the optical stimulation pulses from the photonic probe, a mechanical shutter (Uniblitz TS6B) was used as a blanker during E-pixel illumination pulses. To rule out the potentially confounding effect of the audible “click” generated by this shutter (and heard by the mouse), stimulation pulses were interleaved with control events, in which only the shutter was activated without concomitant light emission [Fig. 5(b)].

In this second proof-of-concept experiment, an individual neuron was reliably activated by light pulses delivered from an implanted photonic probe. Figure 5(d) shows the absence of any response for control events. Analysis of the optical beam trajectory in this experiment indicates that the light emitted by the probe did not impinge upon the cell body of neuron #1 directly, but instead activated a basal dendrite projecting into the center of the beam illumination profile [Fig. 5(g)]. Given the highly collimated nature of light emitted from the probe, simultaneous activation of neighboring neurons—labeled #2, #3, and #4—was not induced. To confirm that these untriggered neurons were indeed activatable, wide-field pulses of blue light illumination were subsequently delivered through the microscope objective. This resulted in widespread, simultaneous activation of the neurons within the illumination field [Fig. 5(f)].

## 8 Discussion

To exploit the full potential of optogenetics, it is essential to deliver light with high temporal and spatial resolution at arbitrary locations within the brain. Here, we demonstrate photonic probes operating at visible wavelengths that permit realization of this goal. Our photonic probes leverage technological developments achieved over the past two decades in the field of optical communications at infrared wavelengths, realizing them within the visible range of relevance for present-day optogenetic reporters and effectors operating at visible wavelengths. Specifically, we employ wavelength division demultiplexing using AWG’s, integrated photonic waveguides, and E-pixels realized as diffractive grating couplers. The technology of visible photonics is rapidly advancing, and this makes it feasible to create a spectrum of components for assembling future complex photonic neural probe architectures. Exceptionally promising candidate technologies will enable fast switching^37^ and lensless beam focusing.^38^ An important attribute of our photonic probe paradigm is their mass producibility via existing photonics foundry protocols. With our achievement of the proof-of-concept reported here, significantly upscaling of E-pixel density and multiplexing is now underway, enabled both with robust and precise foundry-based fabrication protocols and with recent improvements in laser source technology. This will permit their widespread deployment in the near term to the neuroscience and neuromedical research communities.

## Figures and Tables

**Fig. 7:**
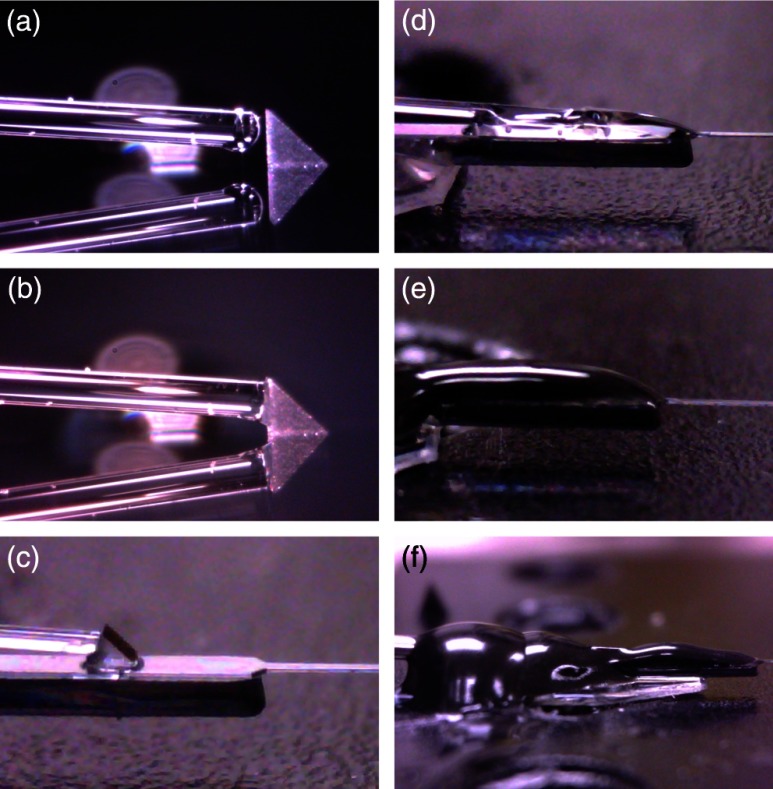
Probe assembly. (a) and (b) Optical fiber is attached to a μ-prism with UV epoxy. (c) Fiber-prism attachment is aligned to the grating coupler and affixed using UV epoxy. (d) and (e) Fiber attachment is secured by applying both additional UV epoxy and medical-grade black epoxy. (f) Image displaying the final packaged probe.

**Fig. 8:**
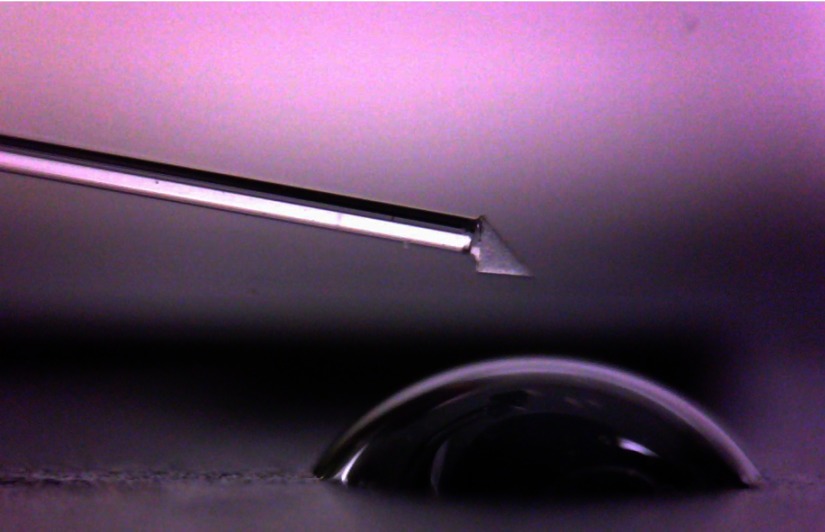
Probe assembly process. Video 1 shows the process by which a small portion of UV epoxy is applied to the bottom surface of the μ-prism. (Video 1, MP4, 24.2 MB) [URL: http://dx.doi.org/10.1117/1.NPh.4.1.011002.1.]

**Fig. 10:**
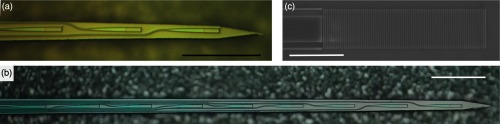
Future probe design. (a) and (b) Photographs showing our next-gen probes, featuring 100  μm spacing between E-pixels. Scale bar represents 100  μm. (c) SEM image showing the next-gen E-pixel design, which has dimensions of 5  μm×20  μm. Scale bar represents 10  μm.

## Appendix A: Probe Fabrication

### A.1 Fabrication Process

Prototype probes were fabricated in Caltech’s Kavli Nanoscience Institute cleanroom facilities. We have developed a fabrication process for silicon nitride (${\mathrm{Si}}_{3}N_{4}$)-based photonic circuits (Fig. 6) that is fully compatible with standard MEMS fabrication techniques. The probes are fabricated on 100-mm SOI wafers, having a top silicon layer thickness of 15  μm (Ultrasil Corporation). This top layer constitutes the structural layer for the shanks and is the dominant contribution to the total shank thickness (18  μm). The optical layers are deposited on top of the silicon structural layer, and comprise a thermally grown 1.5  μm silicon dioxide (${\mathrm{SiO}}_{2}$) layer and a 200-nm stoichiometric ${\mathrm{Si}}_{3}N_{4}$ layer deposited by low-pressure chemical vapor deposition [LPCVD, Fig. 6(a); Rogue Valley Microdevices].

The photonic circuitry is patterned in Ma-N 2403 negative electron-beam resist (MicroChem Corp.), using an electron-beam pattern generator (Leica Microsystems EBPG-5000+). The pattern is then transferred to the ${\mathrm{Si}}_{3}N_{4}$ layer using an inductively coupled plasma (ICP) pseudo-Bosch etch process [Fig. 6(b)]. After stripping the residual e-beam resist, we clad the photonic circuits by depositing a final ∼1  μm ${\mathrm{SiO}}_{2}$ layer using plasma enhanced chemical vapor deposition (PECVD) to complete the optical trilayer [Fig. 6(c)].

Following fabrication of the photonic circuits, we pattern and release the individual probes from the 100-mm wafer. First, we use a liftoff photolithography process, based on AZ 5214E photoresist (Microchemicals, Inc.), to pattern a hard mask into the shape of the probes. The mask is composed of a thin layer of 300-nm-thick aluminum oxide (${\mathrm{Al}}_{2}O_{3}$). Next, a sequence of ICP etch processes is used to etch the probe shape into the front side of the SOI wafer, stopping at the BOX layer [Fig. 6(d)]. Subsequently, a similar process is repeated to etch away the backside of the wafer, leaving only the thin BOX layer framing the probes [Fig. 6(e)]. This layer is then etched away by a quick hydrofluoric acid (HF) dip, which leaves the individual probes anchored to the wafer at four breakable points, and ready for assembly and packaging. Approximately 300 individual probes can be fabricated on a single 100-mm wafer using this fabrication process.

### A.2 Probe Stress Balancing

The design of the probes can be configured to accommodate a wide variety of experiments, with an arbitrary number of shanks, as required. It is critical that the shanks be ideally straight to circumvent buckling during the implantation process or their outright mechanical failure,^28^ as well as to permit stacked assembly of multiple shanks to realize implantable 3-D emitter architectures. This is achieved in our devices by careful balancing of the internal stresses within the probes, through optimization of the ${\mathrm{SiO}}_{2}$ layer thicknesses within the shank. In practice, variations in the fabrication processes within, and between, wafers result in intrinsic stress along the shanks; this can become problematic with increasing shank length. As a proof of concept, we have fabricated shanks with high yield having lengths of 3 and 5 mm; these show minimal stress-induced deflection. We believe that much longer shanks can be achieved using commercial foundry fabrication processes, which achieve superior dielectric layer uniformity and film thickness control for improved stress balancing. Alternatively, for certain applications, it may be possible to increase the shank thickness to achieve the same end.^39^ Our prototypes feature very thin multilayered shanks, with a uniform ∼18-μm thickness along their length [Fig. 1(c)]. This layer thickness is dominated by the silicon structural layer, which provides the primary mechanical strength of the shanks.

### A.3 Probe Packaging

We have developed a dedicated probe assembly and packaging process to attain a lightweight and compact packaged probe footprint (Fig. 7). The optical fiber assembly process is based on coupling to the on-chip integrated photonics elements using a μ-prism, which enables horizontal attachment of the optical fiber to the surface of the probe, rather than vertical attachment. This coplanar orientation of the fiber dramatically reduces the thickness of the packaged probes and greatly improves its mechanical durability.

We have also explored an alternative method for coupling an external optical fiber, also oriented parallel to the chip surface, to an on-chip grating coupler. The second approach utilizes optical fibers that are cleaved at an angle and subsequently polished, and then metalized at the angled ends to create mirrors.^40^^,^^41^ The advantage of using a μ-prism is the flexibility in adjusting the coupling angle between the optical fiber and the grating coupler during the assembly process. The potential optical IL obtained with the approach involving angle-polished fibers can be much lower than the performance attained with μ-prisms, in principle (see section below on IL measurement for details). In practice, however, we find that variations in the fabrication processes obtained in our shared fabrication facility, combined with additional losses due to nonidealities in the input-coupling angle, overwhelm the performance increase obtained using angled polished fibers. However, in the future, our next-generation probes will be fabricated in foundry-based fabrication facilities that can facilitate closely maintained tolerances in coupling angle. In this case, the approach utilizing angled-polished fibers will likely become favorable.

The first step in the packaging process is attaching a bare cleaved optical fiber to a μ-prism. The role of the μ-prism is to transform the propagation direction of the light from the longitudinal to the transverse direction, so it can couple into the grating coupler at almost perpendicular incidence with respect to the grating surface. The fiber is attached to the μ-prism at an angle of 5 deg, which is the designed coupling angle of the grating couplers. The cross section of the μ-prism is only $180\times 180\;\mu m^{2}$ [Fig. 7(a)]. The attachment protocol first involves the application a drop of UV epoxy (Norland Optical Adhesive 85) to the tip of the fiber. This is achieved by dipping the fiber into a drop of epoxy. Then the fiber is aligned to the *μ*-prism within a standard optical fiber alignment setup [Fig. 7(b)]. Finally, the epoxy is cured by illumination with 385 nm UV light from an LED source (Thorlabs). Following attachment of the *μ*-prism to the fiber, the μ-prism is subsequently aligned to the input grating couplers. In this step, the μ-prism is dipped into a drop of UV epoxy, which permits application of a small amount of epoxy to the bottom surface of the μ-prism (Fig. 8). The μ-prism is then aligned using the same setup, and the epoxy is cured with UV light [Fig. 7(c)]. The packaging process is completed by tightly securing the optical fiber to the probe by applying additional UV epoxy (d), followed by medical grade epoxy (e). This provides stress relief for the critical juncture regions between the fiber, μ-prism, and chip. This packaging process (f) results in a compact probe with a total thickness ranging from one to two millimeters.

### A.4 Probe Dimensions

As stated in the main text, using MEMS fabrication techniques, we are able to fabricate implantable shanks, with cross sections of only 20  μm×18 μm at the shank tip. This 18-μm thickness is constant throughout the length of the shank, whereas the shank width grows to 90  μm at the head of the probe. Figure 9 shows a comparison between the dimensions of the shank and the dimensions of a standard 125-μm diameter optical fiber.

As stated in the main text, the E-pixels on our initial prototype probes are spaced 200  μm apart. This spacing is being reduced to 100  μm in our current design (Fig. 10) and will be further reduced to 50  μm, without any requisite changes in the design or fabrication methodology.

## Appendix B: Optical Properties

### B.1 Insertion Loss Measurement

The IL of the waveguides was measured using the cutback method. The ILs of several waveguides of varying lengths were measured and the IL per length was extracted by fitting a curve to data for the IL versus waveguide length. We sampled 36 waveguides, divided into six groups, with increasing lengths spanning from 1 to 11 mm, incremented by 2 mm between groups. Each waveguide was measured by coupling 473-nm blue laser light into the waveguide through an input grating coupler, and subsequently collecting the light emitted by the output grating coupler with a multimode optical fiber (Thorlabs FG050LGA, 0.22 NA). This collected light was detected with an optical power meter (Newport 1936-C). No index matching gel was used. Figure 11 shows the cutback measurement results.

The IL was calculated by fitting the measured results of waveguide transmittance versus waveguide length to an exponentially decaying curve (Fig. 11). We deduce that the IL of these particular waveguides is 13  dB/cm. This value is 10 dB higher than the IL of state-of-the-art foundry fabricated waveguides.^42^ Two major reasons underlie the relatively high losses measured for our waveguides. First, the waveguide sidewalls are rough due to the etching and beam size in the electron beam patterning. Second, random defects are present in the isolating ${\mathrm{SiO}}_{2}$ deposited on top of the waveguides using PECVD. These sources of loss are greatly reduced in commercial photonic foundry processes.

The measured total combined losses of our input and output grating-couplers are, on average, 16 dB. These losses can be understood by considering the details of the grating designs. Both gratings are uniform with straight grating teeth, a designed period of 299 nm, and a duty cycle of 60% (i.e., a 60% etched region per period). A single etch step was employed to define both the waveguides and grating features, but the close proximity and small size of features in the gratings result in the grating teeth having a partial etch of 135-nm depth, whereas the larger waveguides are fully etched [Fig. 11(c)]. This grating design works well for the output grating coupler (E-pixel), which has a simulated IL of 1.8 dB at a wavelength of 473 nm [Fig. 11(d), blue curve]. However, straight grating teeth are not optimal for the input grating coupler, where the input light propagates over roughly 180  μm in the μ-prism and has a curved wavefront;^43^ simulations show that this yields an IL for the input grating of 11.2 dB at 473 nm. Curving the teeth of the input grating coupler could significantly improve the coupling efficiency.^43^ The discrepancy between the 16-dB measured and 13-dB simulated IL of the input and output-grating couplers combined may be due to factors such as reflections at the prism-to-fiber and prism-to-chip interfaces, fabrication variations, and deviation of the input polarization from the optimal, TE polarization assumed in the simulations.

The efficiency of the input grating coupler can be improved by optimizing the thickness of the isolating ${\mathrm{SiO}}_{2}$, and by reducing the propagation distance between the input fiber and the grating. Using in-line fiber coupling with an angle-polished fiber^40^^,^^41^ would shorten the propagation distance to roughly 63  μm and, according to our simulations, will reduce the input IL at 473 nm to 5.8 dB [Fig. 11(d), red curve] and the output IL to 1.7 dB, to a total of 7.5 dB. Further improvement is possible by including a metal back-reflector below the gratings,^44^ which will reduce the amount of light lost into the substrate. Our simulations show this will further reduce the IL to 5.0 dB for the input grating coupler and to 0.7 dB for the output grating coupler. In the future, use of optimized and standardized foundry fabrication^30^ will greatly reduce variations in the design parameters—this is difficult to achieve in shared university fabrication facilities. In foundry-based probes, waveguide propagation losses should be reduced to 2 to 3  dB/cm (corresponding to <1  dB in total for our probes).^42^^,^^45^ Incorporating the above improvements, we project that total optical losses should be <10  dB in our next, foundry-produced, generation of devices.

The typical IL of our AWGs is measured to be 2 to 3 dB. Our IL measurements are carried out using the setup in Fig. 12(c). A supercontinuum laser (WhiteLase micro, Fianium) is used to input broadband light to the AWG, and the transmitted power spectrum through each channel of the AWG is subsequently measured using a spectrometer. A reference measurement is obtained by measuring the transmitted light through a reference waveguide, which bypasses the AWG [Fig. 12(a)]. The excess loss through the AWG is calculated by dividing the spectral power of the former measurement by the latter one.

In these prototypes, the emitting surface area of the E-pixels is 10  μm×10  μm; however, the total footprint of the E-pixel is 100  μm×10  μm to permit incorporation of a tapered waveguide section to optimize coupling between the waveguides and E-pixel’s grating couplers. Improvements to these designs will permit reduction of this footprint to ∼25  μm×10  μm.^46^

### B.2 Illumination Beam Analysis

Characterization of the optical beam profile in a fluorescein–water solution was performed using the measurement apparatus depicted in Fig. 13. The fluorescein solution was prepared by dissolving fluorescein sodium salt (Fisher Scientific LLC) in high (>9.5) pH deionized water to generate a 10-μM concentrated solution. The solution was injected into a thin reservoir composed of two cover slips spaced 1-mm apart (Fig. 13). The probe was inserted sidewise into this fluorescein–water solution and then imaged from above. The inset in Fig. 14 shows an optical micrograph of the measured fluorescein photostimulation response over a distance of 10 mm.

Figure 14 shows simulation results of the beam intensity pattern over the distance spanning 1 mm from the probe surface. According to these simulations, the beam FWHM width at 1 mm is only 30  μm. These results emphasize the exceptional directionality of the illumination pattern generated by the E-pixels.

Figure 15 shows the beam illumination pattern in the first 70  μm, a region where Fresnel difraction dominates the beam profile. High-resolution simulation results of the beam profile in this region qualitatively agree with the measured results. The simulated and measured beam divergence angles in the fluorescent solution are 1.7 and 3.5 deg, respectively. For comparison, this is equivalent to illumination from an optical fiber with a numerical aperture of ∼0.04. Nevertheless, due to the initial strong directionality of the beam, the beam within the brain slice remains tightly confined to a diameter of ∼25  μm, even at a distance of ∼200  μm from the probe surface (Fig. 2).

## Appendix C: Wavelength-Division-Multiplexing

### C.1 Wavelength-Division-Multiplexing Setup

A schematic representation of the optical setup used to address individual channels of the AWG is presented in Fig. 3 of the main text. A supercontinuum laser source (WhiteLase Micro, Fianium) was used as a broadband spatially coherent light source for many of the AWG characterization experiments we conducted. The spectral density of these lasers can be above 1  mW/nm, which is sufficient to excite ChR2 expressing neurons, even when taking into account the probe IL. However, in our wavelength multiplexing demonstration in Fig. 3 of the main text, we used infrared light emitted by an ultrafast (65 fs pulse duration, 80-MHz repetition rate) Ti:Sapphire laser (Tsunami, Spectra Physics) that was converted into blue light using a beta barium borate (BBO) crystal through second harmonic generation process. The converted light had a spectral bandwidth of 5 to 6 nm, a central wavelength of 473 nm, and an average power of 20 mW. A narrow band filter with a nominal FWHM bandwidth of 1 nm (473 OD7 LaserLine Filter, ALLUXA) was used to address individual AWG channels. By manually tuning the incident angle of the laser beam onto the filter, we were able to tune the central passband frequency of the filter.

Figure 16 shows our proposed alternative channel multiplexing design, which can simultaneously control multiple wavelength channels with a short-response time. In this approach, an acousto-optic tunable filter (AOTF) is used to filter narrow-band wavelength channels from the broadband input light. Commercially available AOTFs support up to 10 channels using a single acousto-optic cell^47^^,^^48^ [Gooch & Housego (UK) Ltd., NKT Photonics]; individual channel bandwidths can be as narrow as 0.3 nm. Using several AOTF modules enables tens of channels (E-pixels) to be addressed simultaneously.

### C.2 AWG Design

Our AWG design is based on the detailed theoretical derivation in Ref. 31. The AWGs include one input channel and nine output channels [Fig. 12(d)]. The center channel is positioned at 473 nm and the channel spacing is 1 nm. The overall footprint of the AWGs is $0.02\;{\mathrm{mm}}^{2}$, which is very well suited for integration into our compact neural probe designs.

As explained in the main text, the maximum number of E-pixels that can be addressed by a single AWG is determined by the ratio of the spectral bandwidth of the activation curve of the targeted opsin molecular actuator to the width of an individual AWG spectral channel. However, experimental considerations can further restrict the extent of the opsin activation bandwidth for optimal activation purposes. For example, the targeted brain area might express other opsins with overlapping activation spectra. If crosstalk between two opsins is to be minimized, excitation in the spectral regions in which they overlap should be avoided.

Although, in principle, minimization of the AWG channel bandwidth maximizes the number of addressable E-pixels, in practice, fabrication limitations, optical power, and crosstalk can all reduce the minimum achievable AWG channel bandwidth. Among factors that impose a limit to the achievable reduction of channel bandwidth are fabrication imperfections. Additionally, the spectral density of the power optical emitted from the excitation laser is critical. The power emitted by an individual E-pixel is the laser’s spectral power density integrated over the spectral bandwidth of the channel minus system losses; hence, narrower channels require sources with higher power. Finally, crosstalk between AWG channels can increase with decreasing channel bandwidths.

## Appendix D: Animal Procedures

### D.1 Baylor College of Medicine

All procedures were carried out in accordance with the ethical guidelines of the National Institutes of Health and were approved by the Institutional Animal Care and Use Committee (IACUC) of Baylor College of Medicine. Imaging experiments were performed on ∼6-month old VIP-Cre/ChR2-tdTomato mice (C57Bl/6 background) that were injected 3 to 5 weeks prior to the experiment with 1  μL of a 1∶1 mixture of AAV1-CamKIIa-ChR2(E123T/T159C)-mCherry and AAV1.Syn.GCamp6s.WPRE.SV40 (both viruses from University of Pennsylvania vector core). Injections were performed stereotactically, targeting visual and extra striate cortex ∼300  μm below the surface. Injections were made through a burr hole at a steep (∼60  deg) angle, in order to leave the bone above the transfected region intact, preventing any inflammation of the dura that would obscure the imaging window.

On the day of the experiment, anesthesia was induced with 3% isoflurane and mice were placed in a stereotactic head holder (Kopf Instruments) on top of a homeothermic blanket that maintained their body temperature at 37°C throughout the experiment. Anesthesia was maintained with 1.5% to 2% isoflurane during the surgical procedure. Mice were injected with 5 to 10  mg/kg ketoprofen subcutaneously at the start of the surgery. After shaving the scalp, bupivicane (0.05 cc, 0.5%, Marcaine) was applied subcutaneously, and after 10 to 20 min an $∼1\;{\mathrm{cm}}^{2}$ area of skin was removed above the skull. The wound margins were sealed with surgical glue (VetBond, 3M), and a headbar was attached with dental cement (Dentsply Grip Cement). Throughout the rest of the surgery and experiment, the headbar was used to stabilize the animal’s head. Using a surgical drill and HP 1/2 burr, an ∼3  mm craniotomy was opened above the viral injection site and the exposed cortex was washed with ACSF (125 mM NaCl, 5 mM KCl, 10 mM Glucose, 10 mM HEPES, 2 mM ${\mathrm{CaCl}}_{2}$, 2 mM ${\mathrm{MgSO}}_{4}$).

The anesthetized mouse was positioned under the microscope on a heating pad, and the probe was held in a motorized micromanipulator (Luigs and Neumann). Using the micromanipulator, the probe tip was positioned by eye under the objective at the surface of the cortex. The experiment was then performed under two-photon imaging (920-nm wavelength at 25 to 40 mW, Nikon 16× objective, 0.8 NA). Some unknown characteristic of the probe made the tip visible as a red dot under two-photon imaging, and the probe could also be located by its shadow on the cortex below. The probe was advanced into the cortex through the dura. Typically, some dimpling was observed and the “rebound” of the surface of the cortex indicated that the probe had penetrated through the dura.

Light pulses were delivered from the probe at regular (0.2 to 1 Hz) intervals. Using two-photon imaging of GCaMP6s fluorescence, we searched for and located cells in imaging planes above the probe that appeared by eye to be activated by the light pulses, and recorded their activity for later analysis. The stimulus protocol consisted of two sequentially interleaved conditions. In the first condition, intermittent brief (50 to 400 ms) blue light pulses were delivered through the probe to activate ChR2-expressing cells. Activation in cells coexpressing ChR2 and GCaMP6 was observed as reliable increases (and subsequent characteristic decays) in GCaMP fluorescence following stimulation. During these stimulation periods, a high-speed mechanical shutter (Uniblitz TS1 shutter, ED12DSS controller) protected the PMT in the two-photon microscope from both scattered blue light and one-photon fluorescence from the preparation. Interleaved controls, time intervals where the shutter was closed but no light was delivered, ensured that responses were not being driven by the audible click that the shutter made as it opened and closed.

### D.2 Stanford University

Transgenic Thy1:18-ChR2-EYFP male mice were group housed three to five to a cage and kept on a reverse 12-h light/dark cycle with *ad libitum* food and water. Experimental protocols were approved by Stanford University IACUC and meet guidelines of the National Institutes of Health guide for the Care and Use of Laboratory Animals.

Animals were anesthetized with inhalation of isoflurane (∼1% to 4%) and anesthesia levels were monitored by any overt signs of response to physical stimuli. Once anesthetized, the head was shaved and immobilized in a KOPF stereotaxic apparatus. The animal’s eyes were treated with ointment and the body temperature was maintained by a heating pad. A surgical scrub was done on the skin of the head using betadine and rinsing with 70% ethanol. Next, using sterile instruments, a mid-line scalp incision was made and the scalp was retracted. A small craniotomy (0.5 to 1 mm) over the region of interest was made using a dental drill. Next, the illumination and recording was accomplished by lowering the composite probe to the target location (CA3 of the hippocampus: X=+2.75  mm, Y=−2.54  mm, Z=−2.60  mm, from bregma on the skull). Functional details of the composite probe are described in this paper. Clampex software was used for both recording field signals and controlling the light source of the photonic probe.
